# Considering Factors for Deciding Between Subpectoral and Prepectoral Planes in Direct-to-Implant Breast Reconstruction

**DOI:** 10.3390/jcm15010109

**Published:** 2025-12-23

**Authors:** Tae Hwan Park, Joon Suk Moon, Byeongju Kang, Jeeyeon Lee, Ho Yong Park, Jeong Yeop Ryu, Kang Young Choi, Jung Dug Yang, Ho Yun Chung, Joon Seok Lee

**Affiliations:** 1 Department of Plastic and Reconstructive Surgery, School of Medicine, Kyungpook National University, Daegu 41566, Republic of Koreaprsryu@knu.ac.kr (J.Y.R.); prschoi@gmail.com (K.Y.C.); lambyang@knu.ac.kr (J.D.Y.); hy-chung@knu.ac.kr (H.Y.C.); 2 Department of Surgery, School of Medicine, Kyungpook National University, Daegu 41566, Republic of Korea; joonsukm@gmail.com (J.S.M.); libertas033@gmail.com (B.K.); j.lee@knu.ac.kr (J.L.); phy123@knu.ac.kr (H.Y.P.)

**Keywords:** breast cancer, breast reconstruction, mastectomy, breast implant

## Abstract

**Background/Objectives**: Considering the increasing importance placed on the quality of life among those who survive cancer, breast reconstruction is no longer limited to only compensating for breast loss; achieving the patient’s preferences is now considered. However, the optimal surgical approach (subpectoral plane vs. prepectoral plane) in single-stage direct-to-implant breast reconstruction (DTIBR) has not been established. The aim of this study was to summarize the principles for selecting between the subpectoral and prepectoral planes in DTIBR. **Methods**: In this retrospective study, we evaluated 543 patients with breast cancer who underwent DTIBR between March 2018 and October 2025. Postmastectomy reconstruction was performed in the subpectoral plane when the defect showed greater breast height than width, whereas the prepectoral plane was used when breast width exceeded height. Complications requiring reoperation were analyzed. Patient satisfaction was evaluated based on overall satisfaction, esthetic outcome, physical symptoms, psychosocial impact, and decision satisfaction using a visual analog scale. **Results**: The subpectoral dual-plane approach was most commonly used between 2018 and 2019, while the prepectoral plane became predominant after 2020. Overall, 83 (14.4%) patients developed major complications. The overall satisfaction score was 4.1 ± 0.80 in the subpectoral group and 4.35 ± 0.70 in the prepectoral group, showing a statistically significant difference (*p* value = 0.012). **Conclusions**: The subpectoral and prepectoral planes have distinct advantages and limitations. Ultimately, the reconstructive surgeon should determine the most appropriate option in DTIBR. Selecting the surgical plane based on the postmastectomy defect reduces complications while improving patient satisfaction.

## 1. Introduction

Approximately 2.3 million individuals were diagnosed with breast cancer in 2022 as reported by the World Health Organization, and this malignancy was the most common among women, causing approximately 670,000 deaths. In South Korea, data from the Korea Central Cancer Registry of the National Cancer Center and the Korean Breast Cancer Society show that approximately 35,000 new cases of breast cancer were diagnosed in 2021, accounting for 21.5% of all cases of female cancers and ranking as the most common cancer among women.

Mastectomy is a standard treatment modality for breast cancer, necessitating subsequent breast reconstruction. Reconstructive approaches are broadly classified into autologous tissue transfer and implant-based breast reconstruction. Owing to the ongoing advancements in devices and surgical techniques, implant-based breast reconstruction has recently gained increasing prominence. This reconstruction technique was initially introduced in 1971, using the prepectoral (subcutaneous) plane [1]. However, owing to various complications associated with the procedure, surgical techniques using the subpectoral plane were subsequently developed to minimize adverse outcomes [2,3,4,5]. The introduction of biological materials, such as the acellular dermal matrix (ADM), further advanced the concept, which evolved further into the dual-plane technique [6,7]. Subsequently, in 2001, the concept of one-stage, direct-to-implant breast reconstruction (DTIBR) was first introduced [8]. With the ongoing development of ADM, larger and higher-quality materials have been introduced. Consequently, the concept of positioning the implant in the dual-plane or subpectoral plane to reduce complications has further evolved into a more refined approach—breast reconstruction using the prepectoral plane [9]. Positioning the implant in the original location of the breast parenchyma provides an appropriate anatomical match. Importantly, because this technique does not involve using pectoralis major muscle, muscle-related complications such as animation deformity, pain, and malposition are avoided. However, other complications such as implant visibility or rippling, have been reported in patients with a thin mastectomy flap [10].

The breast is the foremost anatomical structure symbolizing femininity. Therefore, achieving proper bilateral balance, volume, and shape should be considered essential factors, second only to preventing breast cancer recurrence. In contemporary practice, breast reconstruction is no longer limited to compensating for breast loss or restoring the preoperative appearance. Rather, the aim of the process should be to achieve the patient’s desired size, volume, and shape, determined through thorough preoperative consultation and evaluation.

Furthermore, breast surgery is an area within plastic, cosmetic, and reconstructive surgery that most readily reflects evolving trends. Therefore new surgical concepts often emerge and gain widespread adoption over time. However, the focus of this study is not on following such trends, but rather on determining the appropriate surgical technique based on factors such as the breast profile, type of mastectomy, and size and location of the postoperative defect. The primary goal remains the effective treatment of breast cancer and reduction in recurrence rates; however, surgical planning should not be limited to restoring the preoperative breast. The goal should be to achieve an ideal breast shape and volume tailored to each patient. We aimed to summarize and report on the principles underlying such decision-making.

## 2. Materials and Methods

This retrospective study involved 543 patients who underwent DTIBR between March 2018 and October 2025. This study was approved by the Institutional Review Board of Kyungpook National University Chilgok Hospital (IRB No. KNUCH IRB 2025-12-002) The human-derived ADMs used in this study included SC Derm Recon^®^ (DOF Inc., Seoul, Republic of Korea), CGDerm One-Step (CGBio Corp., Seoul, Republic of Korea), CGCryoderm (DGBio Corp., Seoul, Republic of Korea), MegaDerm (L&C Bio, Seoul, Republic of Korea), and Myderm (Biomed, Seoul, Republic of Korea). The ADM thickness ranged from 1–2 mm to 3–5 mm and was selected by the operator based on the thickness of the patient’s mastectomy skin flap. The breast implants utilized in the procedures included Mentor^®^ (Mentor Worldwide LLC, Santa Barbara, CA, USA) and SEBBIN (GROUPE SEBBIN SAS, Boissy l’Aillerie, France) in recent cases. In earlier cases, Natrelle^®^ (Allergan plc, Irvine, CA, USA), Bellagel^®^ (HansBiomed Co., Ltd., Seoul, Republic of Korea), and POLYTECH (POLYTECH Health & Aesthetics GmbH, Dieburg, Germany) were used.

### 2.1. Direct-to-Implant-Based Breast Reconstruction Protocol

The total mastectomy plan was determined by the breast surgeon based on the tumor size and stage, followed by a multidisciplinary combined procedure. In this study, we specifically evaluated patients who underwent breast reconstruction with silicone implants rather than autologous tissue transfer. Before proceeding with direct-to-implant reconstruction, the post-mastectomy skin flap was carefully assessed by the reconstructive surgeon. Reconstruction was performed only when the flap thickness was judged to be adequate, typically ≥1 cm on intraoperative inspection and palpation, and this assessment was incorporated into the intraoperative decision-making process for selecting the reconstructive plane and final implant volume.

**Figure 1:**
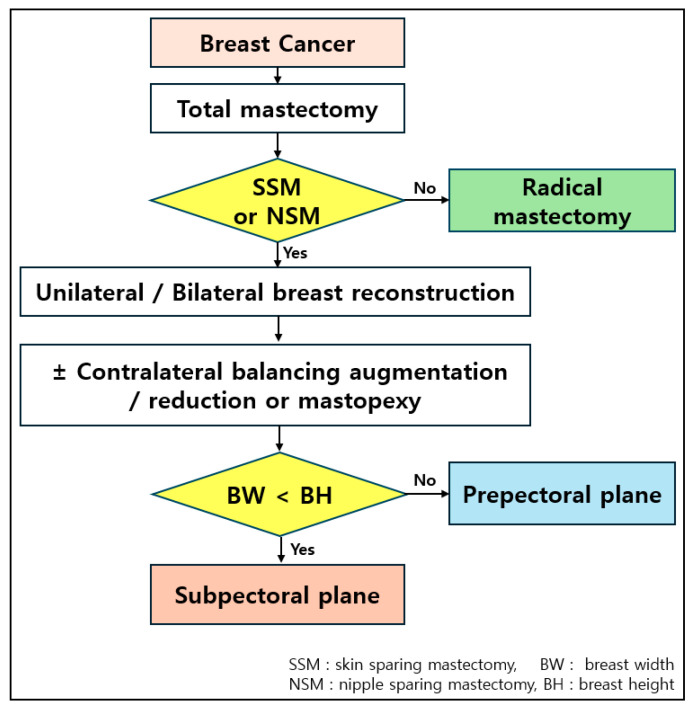
Direct-to-implant breast reconstruction (DTIBR) protocol. In patients requiring total mastectomy for breast cancer, either a skin-sparing or nipple-sparing mastectomy was performed, followed by immediate implant-based breast reconstruction. Augmentation, reduction, or mastopexy may be incorporated as needed, depending on the patient’s breast size and shape. After mastectomy, if the defect showed greater breast height than width, reconstruction was performed in the subpectoral plane. Conversely, when breast width exceeds height, the prepectoral plane was chosen for DTIBR.

First, it was determined whether unilateral or bilateral breast reconstruction was required. Based on this assessment, the desired breast size and shape were evaluated to establish the reconstructive goals and esthetic direction of the procedure. Only the affected breast was reconstructed in unilateral DTIBR performed for therapeutic purposes. When the operation was performed under general anesthesia, the current breast size was reviewed to decide whether augmentation or reduction was required; contralateral breast surgery was performed concurrently to achieve optimal symmetry (Figure 1). This surgery was performed as an esthetic balancing procedure, which was not covered by national health insurance. In patients with bilateral breast cancer, both breasts were reconstructed with DTIBR, prioritizing the patient-desired size and shape to the greatest extent possible. This procedure is covered by national health insurance. All preoperative consultations and intraoperative decisions were made with the goal of achieving the ideal breast size and shape, rather than merely replicating the preoperative breast. The basic method of breast size measurement was illustrated to help the patient visualize and select the desired shape and volume, enabling a more realistic reconstruction and optimal bilateral balance.

### 2.2. Surgical Protocol

**Figure 2:**
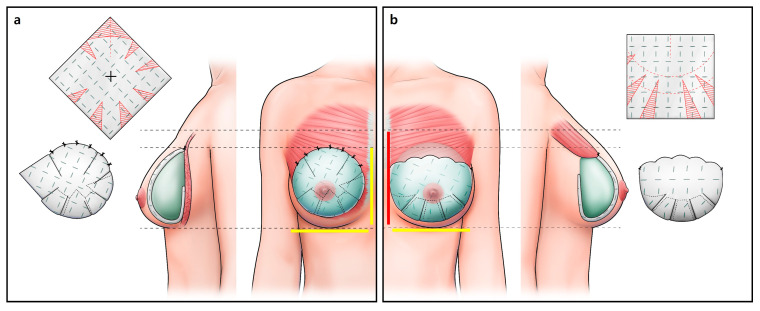
Direct-to-implant breast reconstruction. (**a**) Prepectoral breast reconstruction. Following nipple-sparing mastectomy, when the breast width was greater than or equal to the breast height, a large diamond-shaped acellular dermal matrix (ADM) was designed and positioned in the prepectoral plane. The ADM was radially trimmed to create a pocket that adequately enveloped the implant, allowing for optimal direct-to-implant (DTI) placement. (**b**) Subpectoral breast reconstruction. Following nipple-sparing mastectomy, a rectangular ADM was used if the breast width was smaller than the height. The pectoralis major muscle was adequately released over the upper pole to relieve tension, and the ADM was shaped in an octopus-like pattern to provide smooth, supportive coverage of the lower pole. DTI was then performed in the subpectoral plane. Yellow stick length < red stick length.

Prior to surgery, thorough preoperative consultations were conducted with patients undergoing skin-sparing or nipple-sparing mastectomy (NSM) for breast cancer. During the consultation, the surgical approach using silicone breast implants was explained. The breast size, shape, and degree of ptosis were evaluated, followed by detailed discussions regarding the patient’s current breast contour and desired shape and size. The breast surgeon performed the mastectomy, after which the final reconstructive method was determined based on the shape and size of the resulting defect. After mastectomy, if the defect profile showed a greater breast width than height, reconstruction was planned using the prepectoral plane. Conversely, the subpectoral plane was selected to minimize upper pole depression and rippling complications when the breast height exceeded its width. This decision was not made preoperatively but rather intraoperatively based on the defect profile observed during surgery. Patients were informed in advance of this approach, and consent was obtained accordingly. In patients undergoing reconstruction using the prepectoral plane, a large diamond-shaped ADM was designed by rotating it 45°. A customized ADM was prepared using transverse and vertical incisions to adequately cover a round implant. Depending on the implant projection height, the ADM was trimmed into a series of elongated isosceles triangles at 30–45° intervals to minimize folding and achieve an optimal contour. Through the approach incision made by the breast surgeon, the superior midline of the ADM was initially sutured to the highest point of the defect using 2-0 Vicryl. Additional fixation sutures were subsequently placed bilaterally at approximately 2–3 cm intervals. The upper medial portion of the ADM was then positioned in a rounded configuration to ensure adequate coverage of the subsequently inserted implant. The lower portion of the ADM was evenly spread over the chest wall using long forceps, ensuring that it lay flat without folding or overlapping (Figure 2). The appropriate implant size was determined using an implant sizer while the patient was sitting. The pocket for implant insertion was irrigated with Adams solution (containing cefazolin, among others), after which the ADM was repositioned appropriately. Except for those performed at the upper pole, no additional fixation sutures were placed. The selected implant was inserted using a funnel adopting a no-touch technique. The ADM was then carefully positioned around the implant insertion site near the incision, with particular attention to the lateral aspect. To prevent potential lateral malposition of the implant, 2-0 Vicryl fixation sutures were carefully placed along the lateral margin (Figure 2).

Reconstruction in the subpectoral plane was chosen when the defect height was greater than the width as this approach helped maintain a natural upper pole contour and prevented depression. In robot-assisted NSM, the incision was typically made in the midaxillary area, and thus, the subpectoral plane was often preferred because it offered greater technical ease and accessibility. The lateral border of the pectoralis major muscle was undermined and fully elevated down to its lower margin, followed by partial medial undermining to approximately the midline. This procedure allowed the elevated muscle to provide coverage over the upper one-third or more of the implant to be inserted. To minimize the risk of capsular contracture, malposition, pain, and animation deformity, thorough undermining and adequate muscle release were performed through careful dissection and cutting. A larger rectangular-shaped ADM was subsequently designed, with its superior edge aligned to meet the pectoralis major muscle. This junction was secured with a continuous 2-0 Vicryl suture, which helped minimize postoperative issues, such as palpable knots or suture loosening. The lower portion of the ADM was shaped to provide smooth, supportive coverage beneath the implant, extending medially and laterally to create a natural contour; nevertheless, no fixation sutures were placed in this area. In the sitting position, the appropriately sized implant was inserted with a funnel using a no-touch technique. Two 400 mL drains were placed beneath the implant, one on each side. The incision was subsequently closed in layers using 2-0 and 4-0 Vicryl for the deeper tissues and 5-0 Ethilon or Prolene for the skin. The procedure was then completed (Figure 2).

### 2.3. Implant Selection

During preoperative consultation, the patient’s breast volume, shape, and skin flap thickness were assessed through inspection and palpation. The patient was informed that achieving a fully natural breast contour with a round implant may have certain limitations. Subsequently the patient’s specific esthetic needs and preferences were clarified and addressed via a detailed discussion. If the patient desired augmentation beyond the current breast size, the preferred implant size was determined using illustrations and photographic references. The circumference measurement was used as a general guideline; nonetheless, the patient was informed that this method did not yield an exact surgical correlation. The appropriate implant was then prepared, and the need for surgical intervention on the contralateral, non-diseased breast was thoroughly discussed during the preoperative consultation. Patients were informed that although it was not possible to achieve a perfectly identical reconstruction between the breasts, the goal was to achieve a balanced, symmetrical outcome that did not cause discomfort or functional limitation in daily life (Figure 2). Patients were generally classified into three groups: (1) those who did not wish to undergo augmentation or reshaping but sought reconstruction that restored bilateral volume symmetry when clothed; (2) those who desired implant-based breast reconstruction with volume enhancement and contralateral augmentation for esthetic balancing; and (3) those who preferred reduction in conjunction with implant-based reconstruction because of large breast size. Surgery was performed for all patients after thorough consultation and careful consideration of their individual anatomy and esthetic goals.

### 2.4. Postoperative Complications

In this study, only complications requiring reoperation were analyzed, and the types and incidence rates were evaluated. The major complications were categorized as follows: prolonged seroma or hematoma, implant infection, implant rupture, chronic untreated wound dehiscence, and extensive mastectomy flap necrosis.

### 2.5. Patient Satisfaction

Patient satisfaction was assessed across the following five categories based on the modified KNU Breast-Q, a modified version of the original Breast-Q questionnaire: overall satisfaction, esthetic outcome, physical symptoms, psychosocial impact, and decision satisfaction. A visual analog scale was used, with 5 indicating very satisfied and 1 indicating very dissatisfied [10,11,12]. The survey was conducted at 6 months postoperatively.

## 3. Results

Overall, 543 patients underwent DTIBR, corresponding to 576 reconstructed breasts. The mean age and body mass index were 48.06 ± 8.36 years and 22.23 ± 2.90 kg/m^2^, respectively. Regarding underlying diseases, there were six patients (1.10%) with diabetes mellitus, thirty-two (5.89%) with hypertension, and three (0.55%) with coronary artery disease. In total, 21 patients (3.87%) reported smoking. The mean preoperative breast volume and mastectomy weight were 292.84 ± 134.39 cc and 303.42 ± 183.97 g, respectively. The pathological diagnosis was ductal carcinoma in situ in 175 (30.38%) patients; 352 (61.11%) had invasive ductal carcinoma, 22 (3.82%) had invasive lobular carcinoma, and 14 (2.43%) had lobular carcinoma in situ. Thirteen (2.26%) patients were also diagnosed other malignancies, including malignant phyllodes tumors. Overall, 308 patients underwent NSM, of whom 81 (14.92%) underwent robot-assisted NSM. Skin-sparing mastectomy was performed in 187 patients (32.47%) (Table 1). Regarding the surgical plane, 213 were subpectoral, and 363 were prepectoral reconstructions. The subpectoral dual-plane approach was used in most procedures between 2016 and 2018, whereas the prepectoral plane became predominant after 2018. However, the subpectoral plane showed a gradual resurgence in the most recent 1–2 years. The extent of ADM wrapping also evolved over time. In early prepectoral reconstructions, full wrapping was primarily employed, but this method has progressively been modified into techniques such as the anterior sling (Table 2).

In the early period of implant-based reconstruction, the subpectoral dual-plane technique was predominantly used, with partial ADM placement limited to the lower lateral portion where pectoralis major coverage was insufficient. As larger ADMs became available, surgical approaches gradually transitioned toward the prepectoral plane, accompanied by significant modifications in wrapping techniques.

### Postoperative Outcomes

**Figure 3:**
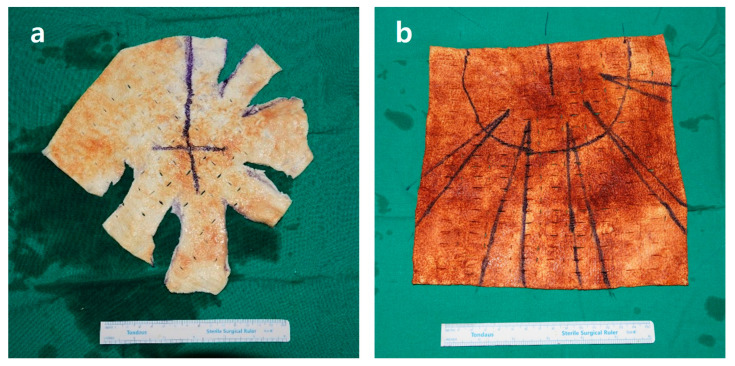
Designs of acellular dermal matrix (ADM). (**a**) ADM configuration for wrapping a round silicone implant in prepectoral DTIBR, showing radial slits to accommodate the lower pole expansion and a central opening for the implant base. (**b**) ADM configuration for subpectoral DTIBR, with a flattened upper margin to allow coverage of the upper pole by the pectoralis major muscle and multiple slits to fit the inframammary fold.

The correlation between preoperative measurements and the postmastectomy defect profiles was analyzed and categorized into five groups. The design of the acellular dermal matrix (ADM) was standardized to appropriately cover a round breast implant, depending on whether the prepectoral or subpectoral plane was selected for reconstruction (Figure 3).

**Figure 4:**
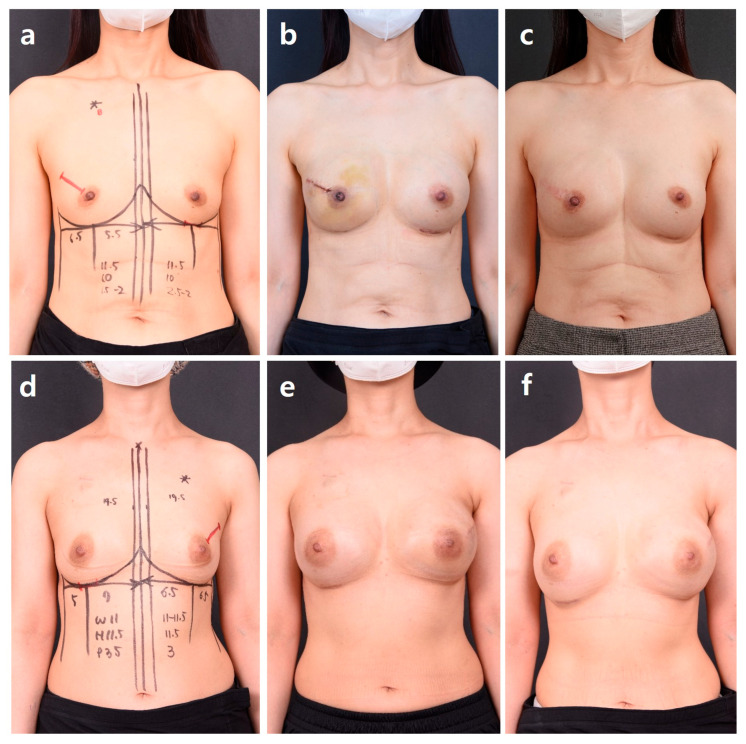
Prepectoral plane selection. Group A: Cases in which the preoperative evaluation and the postmastectomy defect profile are concordant. A 47-year-old woman with cancer of the right breast who has undergone right nipple-sparing mastectomy and contralateral balancing augmentation. Direct-to-implant breast reconstruction was performed on the right side using a Mentor 275 cc smooth round high-profile implant (diameter, 10.8 cm; projection, 4.4 cm), and a Sebbin LSM 210 cc implant (diameter, 11.0 cm; projection, 3.2 cm) was placed in the left breast for esthetic balancing. (**a**) Preoperative findings show breast volume of 180 cc. (**b**) Postoperative findings at 3 weeks. (**c**) Postoperative findings at 6 months. Group B: Cases in which the preoperative evaluation and the postmastectomy defect profile are discordant. A 44-year-old woman with cancer of the left breast who has undergone left nipple-sparing mastectomy and contralateral balancing augmentation. Direct-to-implant breast reconstruction was performed on the left side using a Mentor 330 cc smooth round high-profile implant (diameter, 10.8 cm; projection, 4.4 cm), and a Sebbin Integrity 210 cc implant (diameter, 11.0 cm; projection, 3.2 cm) was placed in the right breast for esthetic balancing. (**d**) Preoperative findings show breast volume of 180 cc. (**e**) Postoperative findings at 1 month. (**f**) Postoperative findings at 6 months.

**Figure 5:**
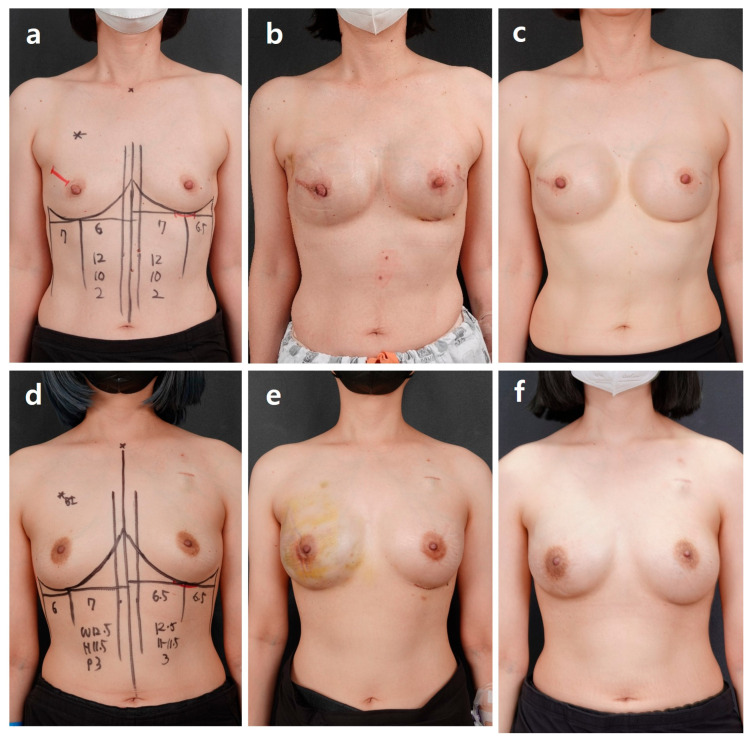
Subpectoral plane selection. Group C: Patients in whom the preoperative breast width was greater than the breast height, but post mastectomy, the defect profile showed breast width smaller than breast height. A 49-year-old patient with cancer of the right breast who has undergone a right nipple-sparing mastectomy and contralateral balancing augmentation. Direct-to-implant breast reconstruction was performed in the right breast using a Mentor 300 cc smooth round high-profile implant (diameter, 10.8 cm; projection, 4.4 cm). A Sebbin Integrity 210 cc implant (diameter, 11.0 cm; projection, 3.2 cm) was placed in the left breast for esthetic balancing. (**a**) Preoperative findings show breast volume of 180 cc. (**b**) Postoperative findings at 2 weeks. (**c**) Postoperative findings at 6 months. Group D: Patients in whom the preoperative breast width is greater than the breast height, but robot-assisted nipple-sparing mastectomy is performed. A 28-year-old patient with cancer of the right breast who completed neoadjuvant chemotherapy and subsequently underwent right robot-assisted nipple-sparing mastectomy with contralateral balancing augmentation. Direct-to-implant breast reconstruction was performed in the right breast using a Mentor 375 cc smooth round high-profile implant (diameter, 12.8 cm; projection, 4.0 cm), and a Mentor Moderate Plus 225 cc implant (diameter, 10.9 cm; projection, 3.3 cm) was placed in the left breast for esthetic balancing. (**d**) Preoperative findings show breast volume of 180 cc. (**e**) Postoperative findings at 2 weeks. (**f**) Postoperative findings at 6 months.

**Figure 6:**
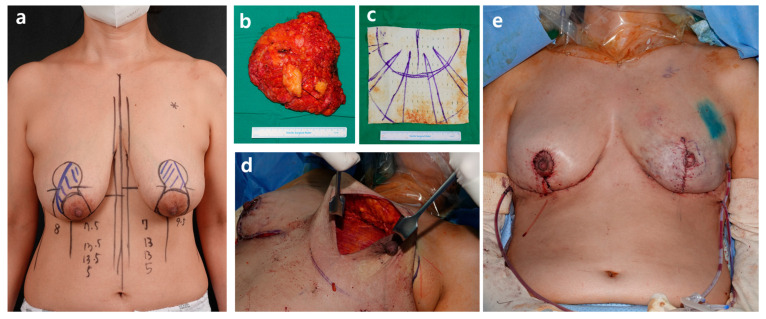
Subpectoral plane selection. (**a**) Preoperative design. (**b**) Excised mastectomy mass (420 g). (**c**) Acelluar dermal matrix design. (**d**) Postmastectomy right breast defect. (**e**) Immediate postoperative findings.

**Figure 7:**
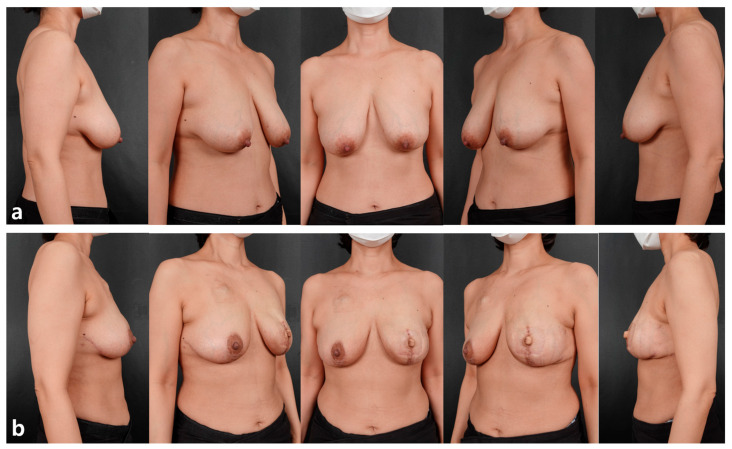
A 48-year-old patient with cancer of the left breast who has undergone a left skin-sparing mastectomy with contralateral balancing reduction mammoplasty. Direct-to-implant breast reconstruction was performed in the left breast using a Mentor 350 cc smooth round moderate-plus profile implant (diameter, 12.5 cm; projection, 3.9 cm), along with simultaneous nipple reconstruction. An inverted-T Wise-pattern reduction mammoplasty was performed in the right breast. (**a**) Preoperative findings showed breast volume of 180 cc. (**b**) Postoperative findings at 3 months.

(1) Group A: Patients in whom the preoperative breast width was greater than its height and the postmastectomy defect profile showed the same relationship (Figure 4).

(2) Group B: Patients with preoperative breast width smaller than its height exhibited the reversed pattern post mastectomy, showing a defect profile of breast width being greater than the height (Figure 4).

(3) Group C: Patients with preoperative breast width greater than the height exhibited a defect profile, post mastectomy, showing breast width smaller than the height; reconstruction was performed in the subpectoral plane (Figure 5).

(4) Group D: For patients with preoperative breast width greater than the height, robot-assisted nipple-sparing mastectomy was performed, and DTIBR was conducted in the subpectoral plane (Figure 5).

(5) Group E: Patients with large or ptotic breasts in whom reduction mammoplasty or mastopexy was concurrently required (Figure 6 and Figure 7).

Major complications were evaluated based on patients who required reoperation. Among the 576 cases, 83 (14.4%) developed major complications, including 12 patients with capsular contracture, 15 with infections, 18 with implant rupture, 12 with skin necrosis, 26 with chronic seroma or hematoma, and 31 requiring simple shape or volume correction (Table 3). Minor complications, including rippling, animation deformity, palpable firm areas, and pain were also observed; however, none required implant removal or additional corrective surgery.

Patient-reported satisfaction was assessed across five domains. Overall satisfaction with breast reconstruction was high (4.2 ± 2.0) and was significantly greater in the prepectoral group than in the subpectoral group. Esthetic outcomes (4.5 ± 1.0) were similarly favorable between groups, with no statistically significant differences detected for any esthetic subscale. Physical symptom scores (4.0 ± 2.0) indicated good tolerance, and patients in the prepectoral cohort reported significantly lower pain levels than those in the subpectoral cohort. Psychosocial satisfaction, including self-esteem-related items (4.2 ± 0.9), was also high, with self-esteem scores being significantly higher in the prepectoral group. Decision-related satisfaction was likewise favorable (4.4 ± 1.3), without a meaningful difference between reconstructive planes (Table 4).

## 4. Discussion

Single-stage DTIBR has been available since 2006. However, there is still no clear consensus among plastic and reconstructive surgeons on whether the subpectoral or prepectoral plane is preferred, as each technique carries its own advantages and limitations [8,13,14,15,16,17]. The prepectoral plane, in which the implant is positioned in the anatomical location of the breast parenchyma, has recently gained attention. Several reports suggest that, when patient selection is appropriate, prepectoral reconstruction may be associated with lower complication rates compared with the traditional subpectoral dual-plane technique [18,19,20,21,22,23]. Moreover, recent studies have reported that even when postmastectomy radiotherapy (PMRT) is performed after DTIBR, there is no significant difference in complication risk between the subpectoral and prepectoral planes. Other studies have also suggested that, with proper patient selection, the quality of life and postoperative complications do not differ significantly between the two groups, and that risks such as animation deformity and rippling can be minimized [24,25,26,27].

**Figure 8:**
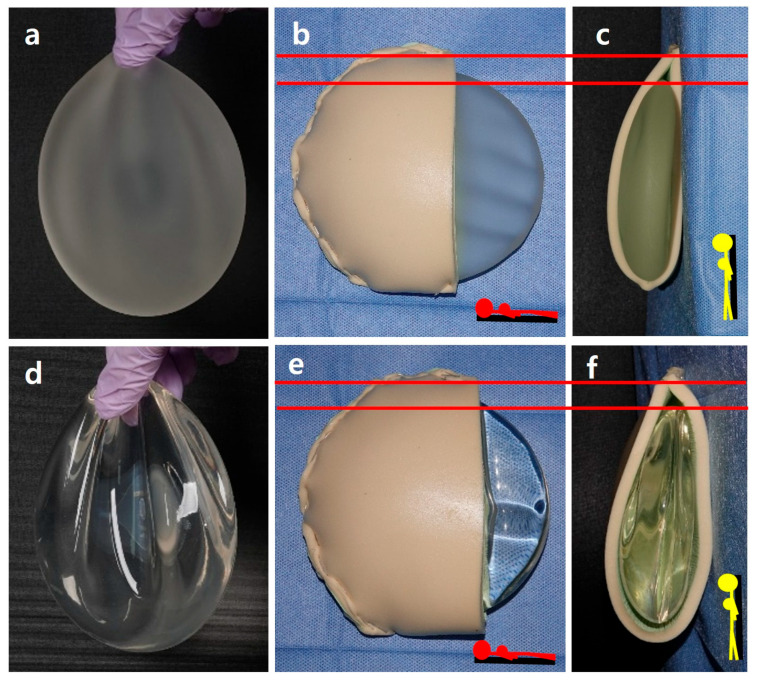
Properties of breast reconstruction implants using phantom models. (**a**) Sebbin Sublimity 76 (semi-moderate, highly cohesive)) 270 cc: base, 112 mm; projection, 32 mm. (**b**,**c**) Supine and upright position in full wrapping model using Sebbin Sublimity 76 and Easyfoam 7 mm. (**d**) Mentor Smooth Round Moderate Classic (MC) Profile 235 cc: diameter, 118 mm; projection, 29 mm. (**e**,**f**) Supine and upright position in full wrapping model using Mentor Smooth Round Moderate Classic Profile 235 cc and Easyfoam 7 mm. Because the implant is made by filling an implant bag with silicone, it is difficult to mimic the natural mesh-like ligamentous structure of the breast parenchyma, and pronounced rippling is observed when the implant is grasped. In the supine position, a small dead space is present, and in the upright position, gravity causes the silicone implant to shift inferiorly, resulting in a slightly larger dead space in the upper area (the red line).

The use of indocyanine green fluorescent agents and related devices to evaluate the condition of the postmastectomy flap has also greatly aided in determining the appropriate surgical approach. Until several years ago, teardrop-shaped textured implants were widely used because they were considered effective in reducing implant malposition and capsular contracture. They were also commonly used for ptotic breast reconstruction and augmentation. However, after reports of breast implant-associated anaplastic large cell lymphoma emerged, these implants were withdrawn worldwide, and practice has shifted back to using smooth round implants [28,29]. Hence, only the projection and filling ratio of implants can be adjusted. Furthermore, although smooth fine implants with fine texture particles have recently been introduced, producing them in a teardrop shape is technically difficult. Accordingly, many reports describing various surgical techniques to accommodate these limitations in implant shape have been published. In addition, various techniques have been reported for covering the implant with ADM. These approaches can generally be divided into two groups: full wrapping and anterior sling. The anterior sling primarily covers the implant’s anterior surface. By performing various techniques and selecting either the subpectoral or prepectoral plane on a case-by-case basis, we have reached several practical, experience-based conclusions, which we present in this report. Though the breast shape, volume, ptosis grade, and profile influence the choice for the type of implant, it is still difficult to determine preoperatively whether the prepectoral or subpectoral plane will be the most appropriate. As described in the Surgical Procedure Section, when using a perfectly circular round implant, it is impossible to adequately restore upper pole volume in patients undergoing postmastectomy with a defect height greater than its width. This is because a round implant cannot provide sufficient upper-pole fullness. Even with thorough preoperative counseling, if upper pole depression or severe rippling develops postoperatively, correction becomes impossible once the capsule has fully formed, unless the implant volume is increased by expanding the breast width to match the breast height. Because an implant is essentially silicone-filled withing a bag, it inherently lacks the mesh-like ligamentous structure of the breast parenchyma. Our phantom model using silicone implants shows that the structural characteristics of the implant inevitably produce rippling, especially when the skin flap is thin, and this occurs depending on posture and position (Figure 8).

In the ADM-covered model, the implant sits within the defect cavity and may appear acceptable in the supine position; however, in the upright position, gravity causes the implant to shift downward, even when the capsule is appropriately formed based on the implant size without contracture. This downward shift creates a superior dead space that cannot be fully eliminated, and it becomes evident that the only way to improve the appearance is through external esthetic adjustments (Figure 8).

**Figure 9:**
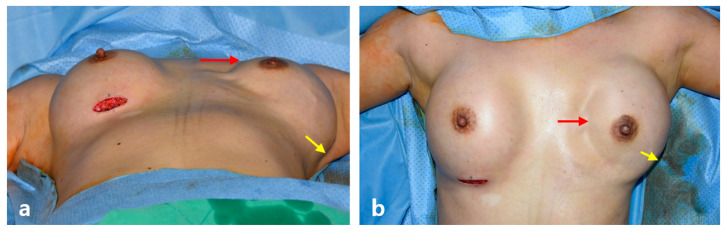
Depression and herniation deformity in a reconstructed breast implant. (**a**) Worm’s eye view. (**b**) Anteropoasterior view. The patient had undergone a left robot-assisted nipple-sparing mastectomy followed by direct-to-implant breast reconstruction. At 1 year postoperatively, the implant was observed to have shifted laterally in the supine position because of the formation of a capsule larger than the implant, resulting in a severe medial depression (red arrow) and lateral herniation (yellow arrow). The defect was corrected using a left mid-axillary robot incision, during which lateral capsulotomy was performed, followed by subcutaneous fixation with 2-0 Vicryl, thereby correcting the deformity. The right breast, which had undergone contralateral balancing augmentation via the inframammary fold approach, showed no malposition-related deformity in the supine position due to the absence of a dead space.

Findings from this phantom model indicate that, although fat injection or ADM filler placement into areas of depression or undulation may immediately improve appearance when rippling occurs, the implant itself remains mobile. In addition, the direction and location of rippling are inherently variable, which limits the overall effectiveness of such interventions. As such, several large ADM materials have recently become available; however, we strongly advise against attempts to fold or stack excess ADM in the upper pole to augment volume. ADM cannot fully simulate the softness of native breast tissue, and when multiple layers are used to add volume, they create even greater firmness. This increased rigidity may result in pain, make the area more palpable, and, in severe cases, may even become visibly prominent, leading to a poor esthetic outcome. These practices have, on rare occasions, also been associated with infection—although not observed in patients who underwent DTI in this series—underscoring why they should be avoided. This approach may be acceptable for patients who desire a breast that remains rigid and fixed—almost like plaster—in the upright position; however, it is not recommended for recreating the breast as an anatomical structure that should retain a degree of softness and natural shape variation with changes in posture. This study shows that by fully elevating the pectoralis major muscle and releasing its inferior margin, the muscle naturally contracts superiorly. This contraction allows it to fill the deficient upper pole of the implant more naturally while also providing a highly satisfying tactile quality. In addition, the method described in this study does not cause complications, confirming its safety and reliability. This is in contrast to the traditional dual-plane subpectoral method, which minimizes ADM use in the lower lateral area and therefore requires limited dissection of the pectoralis major to pull the muscle downward and cover the implant, thereby increasing the incidence of postoperative pain and animation deformity. Furthermore, the naturally contracted pectoralis major provides coverage over the upper portion of the round implant and supplements the upper pole volume, enabling reconstruction that more closely approximates a natural breast shape, with consistently high satisfaction in contour and tactile quality. During surgery, the final stage should be particularly considered, as a capsule that forms larger than the implant in the supine position may allow the implant to herniate laterally toward the mid-axillary area, resulting in severe medial depression of the reconstructed breast (Figure 9).

Therefore, achieving proper lateral fixation between the ADM and the tissues of the lateral margin is essential to obtain a more satisfactory outcome. However, when such complications occur, they can typically be corrected using the original incision approach under local anesthesia, performing a partial capsulotomy and securing the area with 2-0 or 3-0 Vicryl. This technique has consistently produced favorable results. We also recommend considering changing implants to further improve bilateral symmetry. Furthermore, in patients who undergo robot-assisted mastectomy, attempting reconstruction with other techniques has presented challenges. When inserting a fully wrapped implant, the ADM often detaches during placement. Moreover, it is more difficult to properly position the implant after its placement. An alternative approach requires robot-assisted implant-based breast reconstruction. However, this also presents technical challenges and imposes a substantial financial burden on the patient. Therefore, we emphasize the following points. First, determining the reconstruction method preoperatively is challenging, and the choice should instead be made after evaluating the postmastectomy defect. Second, although the preoperative breast shape and volume have been the focus of surgical planning, the demographics of patients with breast cancer have become increasingly younger. Furthermore, with the improved outcomes in early-stage breast cancer, quality of life has become a critical consideration. In addition, as patients’ educational levels continue to improve, there has been a marked increase in individuals who not only prioritize rapid recovery and early return to daily life but also place significant importance on addressing esthetic concerns. In actual preoperative consultations, most Asian patients tend to have small-to-moderate breast volume, and many express that, along with the stress and burden of undergoing total mastectomy and reconstruction for breast cancer, they would prefer to concurrently address long-standing esthetic concerns regarding breast volume or shape. When these issues are corrected concurrently, patients demonstrate markedly higher satisfaction, reflecting improved overall outcomes. Notably, reconstruction with volume enhancement is performed along with contralateral esthetic balancing augmentation for patients with small breasts. Meanwhile, for those with large or ptotic breasts, reduction mammoplasty or mastopexy is incorporated to reduce and reshape the contralateral breast. Most patients diagnosed with bilateral breast cancer experience a modest increase in breast volume rather than excessive augmentation after careful preoperative consultation, although the priority remains treating breast cancer and preventing recurrence. We only recommend surgical approaches that are oncologically safe. A formal clinical study is still being prepared to further substantiate these observations; nonetheless, our current experience indicates that autologous tissue transfer should be recommended for breasts that have already undergone radiation therapy. However, when postmastectomy radiation therapy is planned, single-stage DTI breast reconstruction is preferable over a two-stage approach, as the latter inevitably requires two separate operations and carries a higher risk of complications. Furthermore, radiation has a less significant impact on capsular contracture incidence than on infection incidence, which may reflect advances in radiation-targeting techniques that minimize collateral damage to surrounding tissues.

## 5. Conclusions

The subpectoral and prepectoral planes in DTIBR inherently carry advantages and limitations. Hence, the reconstructive surgeon ultimately determines the most appropriate option on an individual basis. Preoperatively deciding which DTI method to use has clear limitations. Selecting the surgical plane based on the postmastectomy defect reduces complications while improving patient satisfaction. Furthermore, when reconstructive and esthetic goals can be achieved simultaneously, patients are likely to experience an improved overall quality of life.

## Figures and Tables

**Table 1:** Patient characteristics.

Patient Characteristics		Patients	Subpectoral Plane, *n* (%)	Prepectoral Plane, *n* (%)	*p* Value
Patients, *n*/breasts, *n*	Total numbers	543/576	213 (37)	363 (63)	
Mean age	Mean ± SD	48.06 ± 8.36	48.5 ± 8.0	47.8 ± 8.5	0.42
Height, cm	Mean ± SD	159.54 ± 5.49	159.3 ± 5.4	159.7 ± 5.5	0.63
Body weight, kg	Mean ± SD	56.54 ± 7.65	55.8 ± 7.4	57.1 ± 7.8	0.07
BMI	Mean ± SD	22.23 ± 2.90	21.8 ± 2.7	22.6 ± 3.0	0.06
Comorbidities	DM	6	3 (14)	3 (0.8)	0.69
	HTN	32	13 (5.2)	19 (0.7)	0.68
	Coronary a. dz	3	1 (0.5)	2 (0.9)	0.89
	Steroid use	61	24 (11.3)	37 (0.7)	0.67
	Smoking	21	9 (4.2)	12 (0.6)	0.56
Breast volume, cc	Mean	292.84 ± 134.39	298.0 ± 136.0	290.0 ± 133.0	0.39
Mastectomy weight, g	Mean	303.42 ± 183.97	308.5 ± 185.0	300.5 ± 183.0	0.52
Diagnosis	DCIS	175	65 (30.5)	110 (30.3)	0.97
	IDC	352	138 (64.8)	214 (59)	0.18
	ILC	22	7 (3.3)	15 (4.1)	0.63
	LCIS	14	5 (2.3)	9 (2.5)	0.88
	Mucinous carcinoma	7	3 (1.4)	4 (1.1)	0.78
	etc. (fibroadenoma)	6	2 (0.9)	4 (1.1)	0.84
Axillary surgery	Sentinel LN biopsy	498	193 (90.6)	305 (84.0)	0.018
	LN dissection	59	20 (9.4)	39 (10.7)	0.58
	Axillary sampling	19	7 (3.3)	12 (3.3)	0.99
Mastectomy	NSM (no robot)	308	125 (58.7)	183 (50.4)	0.06
	NSM (robot-assisted)	81	35 (16.4)	46 (12.7)	0.63
	SSM	187	53 (24.9)	134 (36.9)	0.09
Contralateral balancing surgery	Augmentation	81	53 (14.6)	28 (13.1)	0.58
	Reduction/mastopexy	19	7 (3.3)	12 (3.3)	0.99
ADM wrapping	Full	89	32 (15.0)	57 (15.7)	0.82
	Partial	487	181 (85.0)	306 (84.3)	0.82
Radiotherapy (RTx)	Postoperative RTx	89	25 (11.7)	64 (17.6)	0.07
	None	476	184 (86.4)	292 (80.4)	0.07
Chemotherapy (CTx)	Neo CTx	91	37 (17.4)	54 (14.9)	0.41
	Adjuvant CTx	93	28 (13.1)	65 (17.9)	0.12
	None	392	148 (69.5)	244 (67.5)	0.63

**Table 2:** History of surgical planes and wrapping methods.

Year		2018–2019	2020–2022	2023–2025
Subpectoral plane		28	43	142
Prepectoral plane	Full wrapping	47	26	16
	Partial wrapping	23	112	139

**Table 3:** Major complications.

Category		Patients, *n* (%) (*n* = 576)	Subpectoral Plane, *n* (%) (*n* = 213)	Prepectoral Plane, *n* (%) (*n* = 363)	*p* Value
Major complications	Capsular contracture	12 (2.2)	5 (2.3)	7 (1.9)	0.91
	Infection	15 (2.8)	5 (2.3)	10 (2.8)	0.62
	Implant rupture	18 (3.3)	6 (2.8)	12 (3.3)	0.59
	Skin necrosis	12 (2.2)	3 (1.4)	9 (2.5)	0.24
	Chronic seroma or hematoma	26 (4.8)	8 (3.8)	18 (5.0)	0.33

**Table 4:** Patient satisfactions using modified KNU Breast-Q. * Statistically significant difference between the two groups (*p* < 0.05).

Category	Questions	Subpectoral Plane (*n* = 213)	Prepectoral Plane (*n* = 363)	*p* Value
**Overall part**	Q1. Overall, are you satisfied with your breast reconstruction?	4.10 ± 0.80	4.35 ± 0.70	0.012 *
**Esthetic part** **(mean ≈ 4.5)**	Q2. Are you satisfied with breast symmetry achieved after reconstruction?	4.45 ± 0.75	4.55 ± 0.70	0.28
	Q3. Are you satisfied with the size of your breast after reconstruction?	4.50 ± 0.70	4.60 ± 0.65	0.22
	Q4. Are you satisfied with the shape of your breast after reconstruction?	4.40 ± 0.80	4.55 ± 0.75	0.18
	Q5. Are you satisfied with the scar resulted after breast reconstruction?	4.35 ± 0.85	4.50 ± 0.80	0.21
**Physical symptoms** **(mean ≈ 4.0)**	Q6. Are you satisfied with how your breasts feel after reconstruction?	4.10 ± 0.85	3.95 ± 0.90	0.19
	Q7. Are you satisfied with the level of pain you had to endure after reconstruction?	3.85 ± 0.95	4.15 ± 0.90	0.008 *
**Psychosocial part** **(mean ≈ 4.2)**	Q8. Have you experienced a loss of confidence or self-esteem after breast reconstruction?	4.35 ± 0.85	4.05 ± 0.90	0.018 *
	Q9. Are you satisfied with your sexual attractiveness after breast reconstruction?	4.25 ± 0.80	4.65 ± 0.65	0.24
**Decisional part**	Total score	4.40 ± 0.55	4.05 ± 0.85	0.091

## Data Availability

The original contributions presented in this study are included in the article. Further inquiries can be directed to the corresponding author.

## Abbreviations

The following abbreviations are used in this manuscript:

ADM	acellular dermal matrix
DTIBR	direct-to-implant breast reconstruction
IDC	invasive ductal carcinoma
ILC	invasive lobular carcinoma
NSM	nipple-sparing mastectomy
DTI	direct-to-implant
